# User and healthcare provider early experiences with the PrEP ring: a quantitative study on the introduction of a new PrEP method in Eswatini

**DOI:** 10.1002/jia2.26490

**Published:** 2025-07-02

**Authors:** Anita Hettema, Siphesihle Shongwe, Haley Sisel, Mxolisi Khumalo, Ncediso Gama, Nolwazi Khanyile, Buyile Mahlalela, Sindy Nana Matse, Jill M. Peterson

**Affiliations:** ^1^ FHI 360 Mbabane Eswatini; ^2^ FHI 360 Durham North Carolina USA; ^3^ Ministry of Health Mbabane Eswatini

**Keywords:** Eswatini, dapivirine vaginal ring, HIV prevention, PrEP choice, women, healthcare provider

## Abstract

**Introduction:**

Eswatini prepared for the national rollout of HIV pre‐exposure prophylaxis (PrEP) choice through a mixed‐method demonstration study introducing the PrEP dapivirine vaginal ring in 12 Ministry of Health‐supported sites. The Eswatini PrEP Ring Study aimed to describe user preferences for, and experiences with, the PrEP ring, and provider impressions. The objective was to provide real‐world data on client preferences and experiences related to PrEP choice, and healthcare provider perspectives on the feasibility and acceptability of offering PrEP choice. A subset of quantitative findings is presented here.

**Methods:**

During May 2023–August 2024, 12 study sites in Eswatini began offering a choice between the PrEP ring and oral PrEP to women 18 years and older who were HIV negative, not pregnant or breastfeeding, and interested in PrEP. Current oral PrEP users who were satisfied with the method were not recruited. Users’ early experiences with the PrEP ring were assessed through structured enrolment and follow‐up questionnaires. Factors associated with choosing the PrEP ring at enrolment were assessed using logistic regression. During the first 2 months of offering PrEP choice, providers (*n* = 16) completed a structured questionnaire on the feasibility and acceptability of PrEP choice service delivery. Clinical Trial Number: NCT05889533

**Results:**

At enrolment, 69% (*n* = 625/904) chose the PrEP ring. Predictors for choosing the ring included ages 25+ (25–34 years AOR = 1.44, 95% CI [1.03, 2.02]; ages 35+ years AOR = 1.69, 95% CI [1.07, 2.68]), higher education (AOR = 1.71 for some/completed high school, 95% CI [1.20, 2.43]; AOR = 1.87 for vocational/tertiary education, 95% CI [1.21, 2.90]) and using either longer‐acting (AOR = 2.23, 95% CI [1.28, 3.89]) or shorter‐acting contraceptives (AOR = 1.63, 95% CI [1.14, 2.32]) versus no modern family planning method. Participants reported high levels of ease and confidence (98%) in using the ring. Ninety‐four percent of PrEP counsellors (*n* = 8) and nurses (*n* = 8) felt prepared to offer PrEP choice and liked choice counselling but had concerns about clients’ ability to return on time for refills of either method.

**Conclusions:**

Many women in Eswatini were willing to try the PrEP ring. Providers were enthusiastic about counselling on PrEP choice and introducing women to the ring.

## 1 INTRODUCTION

The introduction of multiple methods of pre‐exposure prophylaxis (PrEP) for HIV prevention in sub‐Saharan Africa has the potential to increase the accessibility and use of PrEP by allowing individuals, in consultation with their healthcare providers (HCPs), to choose the method that best meets their needs [1].

Available and forthcoming PrEP methods vary by route of administration, efficacy, and personal and clinical considerations, but collectively offer choice for potential end‐users and their HCPs. Daily oral PrEP containing tenofovir disoproxil fumarate is highly effective when taken correctly and consistently, and is recommended by the World Health Organization (WHO) for individuals at substantial risk for acquiring HIV [2]. When used as directed, studies have shown that oral PrEP reduces HIV risk by about 90% or more [3, 4].

The dapivirine vaginal ring (PrEP ring) is a silicone ring inserted vaginally every month that provides a local anti‐HIV effect in the vagina with the non‐nucleoside reverse transcriptase inhibitor dapivirine [2]. In clinical trials, the PrEP ring was found to be approximately 30% efficacious at preventing HIV, with modelling analyses suggesting the effectiveness of about 50% with consistent use in subsequent open‐label studies [5, 6]. The PrEP ring has few side effects and no product‐related serious adverse effects or identified concerns related to HIV treatment resistance [5, 6, 7]. As such, WHO recommends the PrEP ring for women at substantial risk for HIV who cannot or prefer not to use oral PrEP [2].

Injectable cabotegravir, referred to as CAB‐LA, is approved for use in more than 20 countries and has emerged as a promising PrEP delivery mechanism [8].

Oral PrEP was introduced in Eswatini in 2019 and is widely available in the public and private sectors, but national programme data showed that fewer than 40% of eligible people offered oral PrEP adopted it [9]. Early evidence suggests, however, that with additional method options, more people will opt to use PrEP [10]. This reflects what has been seen with family planning (FP), where increasing availability of and access to a range of methods increases overall use [11]. Furthermore, researchers have identified reasons why users in Eswatini have been dissatisfied with oral PrEP and may be seeking other methods, including not wanting to take a daily pill, side effects of oral PrEP, and the stigma of pill taking and its association with HIV [12, 13]. Like many countries in sub‐Saharan Africa, Eswatini recognizes that different users will have different preferences and seeks to offer clients interested in PrEP a choice in PrEP method [14]. Understanding the preferences and experiences of different population groups, including users and HCPs, can guide the development of user‐centred PrEP services that include PrEP method choice.

With support from the Maximizing Options to Advance Informed Choice for HIV Prevention (MOSAIC) project, funded by the President's Emergency Plan for AIDS Relief (PEPFAR) and the United States Agency for International Development (USAID), Eswatini prepared for the national scale‐up of HIV PrEP choice for women through a demonstration study introducing the PrEP ring in 12 PrEP service delivery sites. As CAB‐LA was only introduced in Eswatini at the end of the enrolment period in August 2024, it was not included in our design.

The Eswatini PrEP Ring Study objective was to provide real‐world data on client preferences and experiences related to informed PrEP choice, and HCP perspectives on the feasibility and acceptability of offering informed PrEP choice. This paper summarizes the results of user and HCP experiences with the PrEP ring to provide guidance for key stakeholders in Eswatini in preparation for and during the national programmatic scale‐up of the PrEP ring.

## 2 METHODS

### 2.1 Study design and setting

The study utilized quantitative methods with two populations: a prospective cohort observational design with PrEP users to understand client experiences with PrEP choice and a cross‐sectional quantitative survey with HCPs to assess an enhanced PrEP service delivery package created to enable PrEP choice. The enhanced service delivery package included job aids for the new PrEP options, regular on‐site mentoring, and the integration of PrEP services with broader sexual and reproductive health services. The feasibility and acceptability of PrEP choice were examined from the perspectives of HCPs.

All 12 selected study sites had an established oral PrEP service delivery system and services that catered to adolescent girls and young women (AGYW) and female sex workers, among other populations. Sites were supported by USAID implementing partners and included a mixture of government and private/NGO hospitals, public health units, clinics or outreaches. Eight study sites were fixed sites, while the remaining four were mobile outreach sites. Fifty‐one mobile outreach days at 22 different Determined, Resilient, Empowered, AIDS‐free, Mentored and Safe (DREAMS) locations were counted as a single mobile site for study purposes because they are all managed by one implementer and the same providers attended all mobile outreach days.

Potential participants were approached to enrol in the study during HIV testing and counselling services (HTS) after receiving a negative test result or at other service delivery entry points, including FP and outpatient curative services. If clinically indicated, participants received post‐exposure prophylaxis during the study. Open enrolment continued until the target number of ring initiations (up to 750 based on continuation rates) was achieved, or until 16 months of recruitment elapsed. Prior to study start, HCPs underwent a 1‐day comprehensive training on the use of oral PrEP and the PrEP ring. The training emphasized product‐specific knowledge, counselling, empathy and shared decision‐making to ensure HCPs could offer users informed choice.

### 2.2 Study population

The study involved two populations: cohort participants and HCPs. Potential PrEP‐user cohort participants met the following criteria: HIV negative as per the national HIV testing algorithm on the day of enrolment; female aged 18 and older; not pregnant or breastfeeding; interested in starting PrEP or an existing oral PrEP user interested in the PrEP ring; willing to be contacted for follow‐up; and able to provide written informed consent. Known pregnant and breastfeeding women were excluded due to national guidelines which did not allow for use of the PrEP ring. Existing oral PrEP users were not approached for enrolment unless they asked about different PrEP options or expressed challenges with oral PrEP use.

HCPs, including nurses and HTS counsellors who were involved in PrEP‐related service delivery—including HIV testing and HIV prevention counselling, PrEP initiation, product dispensing or client follow‐up at a site participating in the study—were eligible to participate in the HCP quantitative survey.

### 2.3 Participant recruitment and data collection

Participant recruitment ended in August 2024 with follow‐up data collected until January 2025. During the study, participants were enrolled at the study sites when they visited to receive standard‐of‐care HIV testing. Consenting participants were seen by a trained PrEP HCP and counselled on available PrEP methods. Baseline and follow‐up quantitative surveys were administered by trained data collectors in the participants’ preferred language on the day of enrolment and in follow‐up visits (see File S1). Surveys assessed participant demographics, reasons for choosing their selected PrEP method and early experiences. Participants were contacted by study staff approximately 1 week after enrolment to identify early challenges with their PrEP use.

HCPs completed a structured HCP questionnaire for quantitative data collection conducted at baseline approximately 1−2 months after implementing PrEP choice and the accompanying enhanced service delivery package. Study staff randomly selected two to three PrEP HCPs from each study site for survey completion. The questionnaire adapted items from previously validated measures of intervention acceptability, intervention appropriateness and feasibility of implementation, to describe those elements of PrEP choice service delivery, including the PrEP products themselves and the offering of choice [15]. Similarly, as part of the survey, data collectors solicited HCPs’ concerns about each PrEP method and asked them to reflect on the PrEP choice training and preparation provided.

### 2.4 Data analysis

A descriptive analysis of cohort and HCP questionnaires was conducted in Stata 18 [16] and summarized using frequencies and percentages. To answer our study objective to describe user preferences for the PrEP ring, we used logistic regression to explore factors associated with choosing the PrEP ring over oral PrEP at enrolment. For categorical variables, the reference group was chosen as the lowest value or level of the variable (e.g. youngest age category). All participants who chose a method at enrolment and had non‐missing demographic information (*n* = 848) were included in the logistic regression. Covariates with a *p*‐value of ≤ 0.1 in bivariable models were candidates for the multivariable model, where statistical significance was determined using a *p*‐value of < 0.05. For categorical variables with more than two levels and an overall significant effect determined by Chi‐squared tests (*p*‐value ≤ 0.1), all levels of the covariate were included in the adjusted model, and individual comparisons to the reference group were made. Crude and adjusted odds ratios (ORs) with 95% confidence intervals (CI) were used to describe the strength of the association. Chi‐squared tests were used to further explore associations.

### 2.5 Ethical considerations

The study protocol was reviewed and approved by the FHI 360 Protection of Human Subjects Committee and the Eswatini Health and Human Research Review Board prior to study implementation. Written informed consent was obtained from both cohort and HCP participants in the subject's preferred language prior to participation. Neither cohort members nor providers were compensated for their time. While no serious adverse events occurred, safety monitoring procedures were in place to report and document any adverse reactions or social harms related to study participation. The study was registered under Clinical Trial Number: NCT05889533.

## 3 RESULTS

### 3.1 Cohort participant characteristics and factors associated with choosing the PrEP ring at enrolment

From May 2023 through August 2024, 904 participants enrolled in the study and received choice counselling for oral PrEP and the PrEP ring. Most participants were under 35 years old (83%), single (72%) and had completed at least some high school, vocational or tertiary education (61%) (Table 1). Although 57% of participants were PrEP naïve at enrolment, 34% were previous oral PrEP users (had not used in the 30 days prior to enrolment).

**Table 1:** Participant demographics at study enrolment

	Method choice at enrolment			
	**Oral PrEP**	**PrEP ring**	**No method**	**Total**
	***n* = 248 (27%)**	***n* = 625 (69%)**	***n* = 31 (3%)**	***N* = 904 (100%)**
**Age**, mean (SD)	27.1 (7.3)	28.3 (7.0)	26.6 (6.6)	27.9 (7.1)
**Age**				
18–24 years	112 (45%)	221 (35%)	15 (48%)	348 (39%)
25–34 years	101 (41%)	287 (46%)	11 (36%)	399 (44%)
35+ years	35 (14%)	117 (19%)	5 (16%)	157 (17%)
**Highest education at enrolment**				
Secondary or less	108 (44%)	218 (35%)	2 (7%)	328 (36%)
Some or completed high school^a^	84 (34%)	254 (41%)	8 (26%)	346 (38%)
Vocational or tertiary	51 (21%)	136 (22%)	14 (45%)	201 (22%)
Missing/unknown	5 (2%)	17 (3%)	7 (23%)	29 (3%)
**Relationship status at enrolment **				
Single/divorced/separated/widowed	185 (75%)	437 (70%)	27 (87%)	649 (72%)
Married/partner	63 (25%)	188 (30%)	4 (13%)	255 (28%)
**Prior PrEP use at enrolment **				
PrEP naïve	148 (60%)	345 (55%)	22 (71%)	515 (57%)
Current PrEP user (≤30 days)	20 (8%)	65 (10%)	1 (3%)	86 (9%)
Previous PrEP user (>30 days)	80 (32%)	215 (34%)	8 (26%)	303 (34%)
**Number of sexual partners in 3 months prior to enrolment**				
≤1	221 (89%)	23 (74%)	23 (74%)	787 (87%)
≥2	27 (11%)	7 (23%)	7 (23%)	116 (13%)
Missing/unknown	–	1 (3%)	1 (3%)	1 (0.1%)
**In a known sero‐different relationship with primary sex partner at enrolment** ^b^				
No	219 (88%)	565 (90%)	28 (90%)	812 (90%)
Yes	29 (12%)	60 (10%)	3 (10%)	92 (10%)
**Modern contraceptive use at enrolment**				
No FP (including withdrawal)	92 (37%)	175 (28%)	13 (42%)	280 (31%)
Longer acting (implant, IUD, BTL)	21 (9%)	85 (14%)	2 (7%)	108 (12%)
Shorter acting (injectable, pill)	103 (42%)	307 (49%)	8 (26%)	418 (46%)
Other FP (emergency contraception, condoms)	29 (12%)	58 (9%)	5 (16%)	92 (10%)
Missing/unknown	3 (1%)	–	3 (10%)	6 (1%)
**Service delivery place **				
Fixed site	196 (79%)	547 (88%)	30 (97%)	773 (86%)
Mobile clinic	52 (21%)	78 (12%)	1 (3%)	131 (14%)

*Note*: Percentages may not sum exactly to 100% due to rounding.

Abbreviations: BTL, bilateral tubal ligation; FP, family planning; IUD, intrauterine device; SD, standard deviation.

^a^ In Eswatini, high school consists of 2 years of education beyond the 4 years of secondary school.

^b^ The 544 participants who did not know the HIV status of their last sex partner or did not answer this question were coded as not in a *known* sero‐different relationship.

The mean age of participants at enrolment was 27.9 (IQR 23.0–32.0); most participants (*n* = 625; 69%) opted for the PrEP ring. Participants cited diverse reasons for choosing the PrEP ring, including ease of use (57%), no need for a daily pill (56%), effectiveness (16%) and ability to use discreetly (13%). Factors statistically significant in their association with choosing the PrEP ring at enrolment in the adjusted model included being aged 25 or older compared to those 18–24 years (25–34 years AOR = 1.44, 95% CI [1.03, 2.02], *p* = 0.031; 35+ years AOR = 1.69, 95% CI [1.07, 2.68], *p* = 0.025); having higher education compared to those with secondary education or less (up to high school, AOR = 1.71, 95% CI [1.20, 2.43], *p* = 0.003; vocational or tertiary, AOR = 1.87, 95% CI [1.21, 2.90], *p* = 0.005); and using longer‐acting contraceptives (implant, intrauterine device [IUD], bilateral tubal ligation [BTL]) (AOR = 2.23, 95% CI [1.28, 3.89], *p* = 0.005) or shorter‐acting FP methods, including the injectable or oral pill (AOR = 1.63, 95% CI [1.14, 2.32], *p* = 0.008), versus no FP (Table 2). In addition, those enrolled at mobile sites had lower odds of choosing the PrEP ring than those enrolled at fixed sites, after adjusting for age, highest education level and contraceptive method (AOR = 0.60, 95% CI [0.39, 0.91], *p* = 0.016). Most sites (*n* = 11/12) experienced stockouts of oral PrEP, which is provided by the Ministry of Health (MOH), during the study. Stockouts lasted 2−49 days in length and affected 78 individuals who could not be dispensed oral PrEP at enrolment. In sensitivity analyses with periods of known national oral PrEP stockouts removed, the proportion of study participants choosing the PrEP ring at enrolment remained high at 67% (*n* = 552/826).

**Table 2:** Logistic regression model of predictors of choosing PrEP ring at enrolment

Covariate	*N* = 848	Crude OR	*p*‐value	Adjusted OR [95% CI]	*p*‐value
	*n* (%)	[95% CI]			
**Age**					
18–24 years	327 (39%)	REF		REF	
25–34 years	373 (44%)	1.53 [1.10, 2.12]	**0.011**	1.44 [1.03, 2.02]	**0.031**
35+ years	148 (18%)	1.66 [1.07, 2.58]	**0.025**	1.69 [1.07, 2.68]	**0.025**
**Highest education at enrolment**					
Secondary or less	325 (38%)	REF	**0.022**	REF	**0.003**
Some or completed high school^a^	338 (40%)	1.48 [1.06, 2.08]	0.13	1.71 [1.20, 2.43]	**0.005**
Vocational or tertiary	185 (22%)	1.36 [0.91, 2.03]		1.87 [1.21, 2.90]	
**Relationship status at enrolment**					
Single/divorced/separated/widowed	601 (71%)	REF	0.136		
Married/partner	247 (29%)	1.29 [0.92, 1.81]			
**Prior PrEP use at enrolment **					
PrEP naïve	479 (56%)	REF	0.287		
Current PrEP user (≤30 days)	83 (10%)	1.34 [0.78, 2.30]	0.386		
Previous PrEP user (>30 days)	286 (34%)	1.16 [0.83, 1.60]			
**Number of sexual partners in 3 months prior to enrolment **					
≤1	741 (87%)	REF	0.326		
≥2	107 (13%)	1.27 [0.79, 2.02]			
**In a known sero‐different relationship with primary sex partner at enrolment **					
No	759 (90%)	REF			
Yes	89 (10%)	0.80 [0.50, 1.28]			
**Modern contraceptive use at enrolment**					
No FP (including withdrawal)	261 (31%)	REF	**0.007**	REF	**0.005**
Longer acting (implant, IUD, BTL)	104 (12%)	2.12 [1.23, 3.64]	**0.006**	2.23 [1.28, 3.89]	**0** **.008**
Shorter acting (injectable, pill)	397 (47%)	1.61 [1.15, 2.27]	0.846	1.63 [1.14, 2.32]	0.865
Other FP (emergency contraception, condoms)	86 (10%)	1.05 [0.63, 1.76]		1.05 [0.62, 1.77]	

*Note*: Percentages may not sum exactly to 100% due to rounding. The bold values are *p*‐values that are statistically significant.

Abbreviations: BTL, bilateral tubal ligation; CI, confidence interval; FP, family planning; IUD, intrauterine device; OR, odds ratio; REF, reference.

^a^ In Eswatini, high school consists of 2 years of education beyond the 4 years of secondary school.

### 3.2 Ring insertion at enrolment

Ring insertion by cohort participants or with support from an HCP was encouraged at the enrolment visit. A total of 361 (58%) participants inserted the ring at enrolment. Of these, the majority (86%) self‐inserted and only a small proportion (14%) requested HCP support. Ring insertion was highly dependent (*p* < 0.001) on the type of site, with 65% of ring users enrolled at fixed sites leaving with the ring inserted compared to only 12% of those at mobile clinics (Table 3).

**Table 3:** Ring insertion at enrolment

	Fixed sites (*n* = 547)	Mobile clinic (*n* = 78)	Total (*N* = 625)
	***n* (%)**	***n* (%)**	***n* (%)**
Self‐inserted at enrolment	310 (57%)	2 (3%)	312 (50%)
HCP supported insertion at enrolment	42 (8%)	7 (9%)	49 (8%)
Not inserted at enrolment	195 (36%)	69 (88%)	264 (42%)

*Note*: Percentages may not sum exactly to 100% due to rounding.

Abbreviation: HCP, healthcare provider.

When contacted approximately 1 week after enrolment, 87% (200/230) of cohort participants who had not inserted the ring at enrolment self‐reported successful ring insertion. The 30 individuals who had not yet inserted the ring 1 week after enrolment commonly cited being on their menstrual period (*n* = 8), undergoing sexually transmitted infection treatment (*n* = 3), being afraid of side effects (*n* = 3) or being generally afraid to insert the ring (*n* = 3). Among the 332 participants who left the enrolment visit with the ring inserted and who were reached 1 week later, 28 (8%) reported that they had removed the ring (at least temporarily) because it felt uncomfortable (*n* = 8) or they had had side effects (*n* = 9) including abdominal pain (*n* = 6) and vaginal burning/itching (*n* = 2), or because it fell out (*n* = 10).

### 3.3 Return rates and method choice at first follow‐up visit among ring users

Fifty‐one percent of participants who chose the PrEP ring at enrolment returned for at least one follow‐up appointment (319/625) (median 29 days after enrolment) including 224/625 (36%) who returned “on time,” defined in Eswatini as within 35 days of their enrolment visit. The other 95 participants (15%) returned an average of 92 days (median 72 days) after enrolment. Among the 165 who did not return for a follow‐up visit and provided a reason for the missed appointment, the most common reasons were: difficulties returning, including transportation challenges; away from home and travelling (34%); too busy or work/school obligations (22%); and discontinuing the PrEP ring because of side effects (15%).

**Figure 1:**
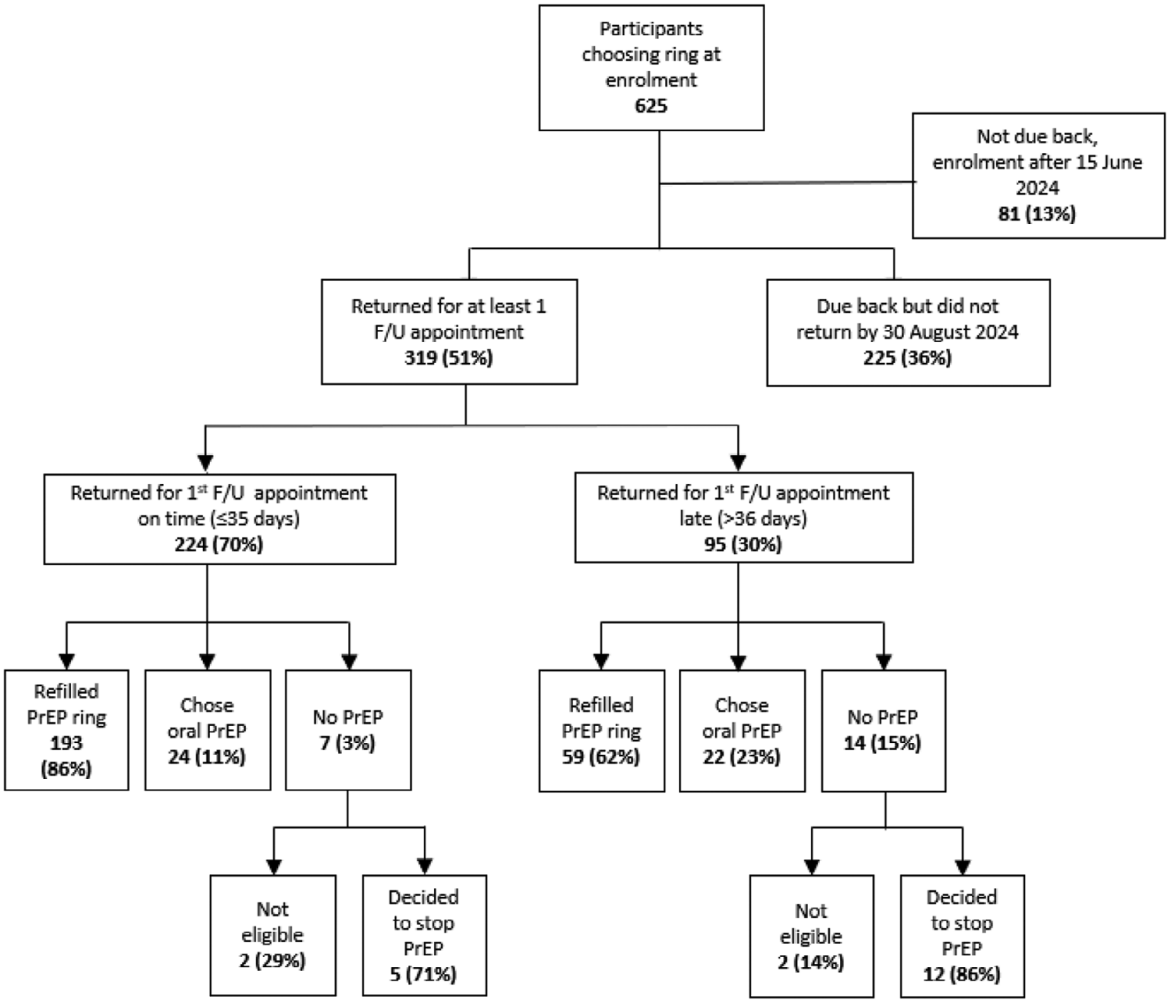
**Ring user flow diagram**. Abbreviations: F/U, follow‐up; PrEP, pre‐exposure prophylaxis.

Seventy‐nine percent (252/319) of those who chose the ring at enrolment and returned for a follow‐up visit also chose the ring at their first follow‐up (Figure 1). Outcomes for the remaining returning participants can be seen in Figure 1.

### 3.4 Ease of ring use among participants selecting ring at enrolment

Most ring users mentioned at their first follow‐up appointment that it was somewhat easy (*n* = 32/252; 13%) or very easy (*n* = 213/252; 85%) to use the ring and were confident (*n* = 78/252; 31%) or very confident (*n* = 169/252; 67%) they would be able to continue using the ring as prescribed by their HCP.

### 3.5 HCP demographics and perceptions of PrEP choice

Sixteen HCPs from seven fixed sites participated in the HCP survey, including seven registered nurses, one nurse assistant and eight HTS counsellors (Table 4). One fixed site was excluded due to its later study activation. Mobile sites were excluded due to an overlap in HCP staff with fixed sites.

**Table 4:** Demographics of healthcare providers responding to the quantitative survey

	*N* = 16
	*n* (%)
**Age**	
25–34 years	6 (38%)
35–44 years	6 (38%)
45+ years	4 (25%)
**Sex**	
Female	14 (88%)
Male	2 (12%)
**Designation**	
Registered nurse	7 (44%)
Nurse assistant	1 (6%)
HTS counsellor	8 (50%)
**Years of experience in role**	
1–4 years	6 (38%)
5–9 years	8 (50%)
>10 years	2 (13%)
**Years of experience in providing PrEP**	
<1 year	2 (13%)
1–3 years	9 (56%)
>3 years	5 (31%)

*Note*: Percentages may not sum exactly to 100% due to rounding.

Abbreviations: HTS, HIV testing services; PrEP, pre‐exposure prophylaxis.

### 3.6 Perceptions of PrEP choice delivery and preparedness

All HCPs (*n* = 16) responding to the quantitative survey believed that counselling AGYW on sexual and reproductive health was part of their job and that information on PrEP should be included in HIV prevention education for AGYW. Nearly 90% of survey respondents felt “very prepared” to offer PrEP choice counselling, with three‐quarters reporting they had received sufficient clinical training and information to offer oral PrEP and 56% to offer the PrEP ring.

Nearly all quantitative respondents (94%) reported that they liked offering PrEP choice counselling to clients and believed offering PrEP choice was a good match with client needs. Ninety‐four percent also thought it was possible to offer the service at their site.

### 3.7 Barriers to and concerns about offering PrEP choice

Concerns about the lower efficacy of the ring compared to oral PrEP were mentioned by 94% of HCPs in the quantitative survey. Clients’ ability/willingness to insert and remove it on their own was also a concern for 63% of survey respondents.

Ninety‐four percent of HCPs were concerned about clients’ ability to return on time for resupply for both oral PrEP and the PrEP ring, with 81% worried about clients’ ability to follow a daily oral PrEP schedule.

## 4 DISCUSSION

Study findings suggest that interest in the ring was high among women in Eswatini who were PrEP naïve, had not used PrEP in 30 days or were dissatisfied with oral PrEP. Interest was particularly high among women ages 25 or older and those who had higher levels of education.

The ring was more popular at enrolment among those reporting using modern FP methods, such as implants, IUDs, oral pills or injectable contraception. This popularity among contraceptive users may be the result of study recruitment taking place through FP services. Preference for the PrEP ring over oral PrEP may be the result of users being partial to methods that do not require daily adherence; the oral contraceptive pill is used by only 12% of modern FP users in Eswatini, whereas injectable contraception is used by nearly 32% [17]. Other research has shown that preferences for PrEP methods may be correlated with specific FP methods currently or previously used [18, 19]. Lastly, FP users who experience side effects from their FP method may have been more likely to choose the PrEP ring over oral PrEP because they wanted to limit further side effects [5].

The reasons for lower interest in PrEP ring at mobile sites warrant further research, but privacy and/or space issues may have contributed. Some mobile sites were located near factories to target staff, but employees may have had limited time for appointments and insufficient time or privacy to insert the PrEP ring themselves during visits.

Ring insertion at the time of enrolment was more common with those enrolling at fixed sites. As Inghels et al. emphasize, however, clients have varying preferences for PrEP delivery settings, indicating the need for multiple service delivery models to enhance accessibility and uptake [20]. The MOH in Eswatini has emphasized their preference that clients self‐insert to build client confidence, reduce provider workload and free up service delivery spaces [21].

Only approximately one‐third of ring clients returned for a follow‐up visit within 35 days of enrolment. Although this may be due to dissatisfaction with the method, including side effects or no longer having a need for PrEP, many participants had difficulties returning for a follow‐up visit. This underscores the importance of investing in activities that enable longer PrEP use such as longer refill periods, community‐based PrEP distribution and improved follow‐up to manage side effects [22, 23, 24].

Additionally, multi‐month dispensing of the PrEP ring would reduce appointment burdens on both clients and HCPs, and allow clients to align PrEP and long‐acting contraception visits [25]. A recent study by Ngure et al. found that clients given 6 months of oral PrEP had similar HIV testing, retention and adherence outcomes as those on the standard‐of‐care 3‐month PrEP dispensing schedule (with quarterly clinic visits) [24]. Six‐monthly dispensing for oral PrEP and the PrEP ring has recently been approved in Eswatini's national guidelines [21].

Not only were clients enrolled in the cohort enthusiastic about PrEP choice, but HCPs liked to offer it to their clients. They felt it was a good match to client needs, and feasible to offer at their sites. This echoes previous research in Eswatini by Barnighausen et al., who found that healthcare workers had adapted their practices to better deliver PrEP in primary care settings, demonstrating flexibility and responsiveness to client needs [26]. HCPs concerns regarding product efficacy reflect findings from other research, including clinical trials of the PrEP ring and oral PrEP, where low adherence has been associated with lower efficacy [5, 6, 27, 28]. Provider concerns about clients’ ability to insert and remove the ring on their own, however, did not reflect related research, where participants have reported that inserting the ring was “not difficult at all” and got easier over time [29, 30]. These concerns were also not reflected in the results from the ring‐user cohort survey, where most study participants reported that the ring was easy to use and felt confident about using it in the future.

### 4.1 Limitations

Selection bias for cohort participants in this study was likely influenced by several factors, including that oral PrEP is available in the public sector in Eswatini, whereas the PrEP ring was only available through participation in the study. In addition, current oral PrEP users were not approached to enrol in the study unless they had mentioned dissatisfaction with oral PrEP to the HCP. Occasional national stockouts of oral PrEP led the PrEP ring to be the only option for several months. When we controlled for periods of national stockout of oral PrEP, however, the percentage of choosing rings was not statistically different. Feedback on PrEP ring use was gathered only from those who returned and thus could over‐represent those whose experiences were more positive. Our sample sizes for providers were limited, as some sites have few HCPs, which may reduce the robustness of our findings.

## 5 CONCLUSIONS

The introduction of new PrEP methods in sub‐Saharan Africa represents a significant advancement in HIV prevention. The Eswatini PrEP Ring Study shows that many women are open to a new method and HCPs are enthusiastic about counselling on PrEP choice and initiating women on the PrEP ring. The study highlights the diversity in client needs and that no single product will meet the needs of all women or potential PrEP users. Follow‐up with clients to support their ongoing PrEP use is important.

## COMPETING INTERESTS

The authors have no competing interests to disclose.

## AUTHORS’ CONTRIBUTIONS

AH, SS, HS and JMP conceptualized the manuscript. Quantitative data analysis was conducted by MK, HS and NG. All authors contributed to the writing, review and approval of the manuscript.

## FUNDING

This manuscript is made possible by the generous support of the American people through the U.S. President's Emergency Plan for AIDS Relief (PEPFAR) and the United States Agency for International Development (USAID).

## DISCLAIMER

The contents are the responsibility of the Maximizing Options to Advance Informed Choice for HIV Prevention (MOSAIC) project and do not necessarily reflect the views of PEPFAR, USAID or the U.S. Government. MOSAIC is a global cooperative agreement (Cooperative Agreement 7200AA21CA00011) led by FHI 360, with core partners Wits RHI, Pangaea Zimbabwe, LVCT Health, Jhpiego and AVAC.

## Supporting information



Supportive information
**File S1**: Eswatini Ring Study Sample survey questions.
A pdf file with sample questions for the enrolment, week 1 phone call and follow‐up surveys.

## Data Availability

As some study indicators beyond those reported here are not yet final, de‐identified data for the study will be made publicly available following study completion through an open‐data platform. Further inquiries can be directed to the corresponding author.
