# Supplementary material for: User and healthcare provider early experiences with the PrEP ring: a quantitative study on the introduction of a new PrEP method in Eswatini
Source: J Int AIDS Soc. 2025 Jul 2;28(Suppl 2):e26490. doi: 10.1002/jia2.26490 (PMC12215817; doi:10.1002/jia2.26490)
Supplement: Supplementary file 1 — Supportive information File S1: Eswatini Ring Study Sample survey questions. A pdf file with sample questions for the enrolment, week 1 phone call and follow‐up surveys. [file JIA2-28-e26490-s001.pdf]

Eswatini Ring Study_Sample survey questions

Sample enrollment questions:

Number	Question	Response options
101	Age (in completed years)	
101.b	What is the highest level of school you have attended?	None<br>Some Primary (Grade 1 to 6)<br>Completed Primary (Finished Grade 7)<br>Some Secondary (Form 1 to Form 2)<br>Completed Secondary (Finished Form 3)<br>Some High school (Form 4)<br>Completed High school (Finished Form 5)<br>Some vocational<br>Completed vocational<br>Some tertiary<br>Completed tertiary<br>Don't know<br>No response
101.c	Are you currently in school?	Yes<br>No<br>No response
102	Relationship status	Single<br>Married/ cohabiting<br>Divorced/ separated<br>Widowed<br>Other (specify)<br>Don't know<br>No response
103	Who do you live with currently?	Partner<br>Parents<br>Other family<br>Friends<br>Alone<br>Other (specify: ________)<br>Don't know<br>No response
104	Religion	None<br>Catholic<br>Anglican<br>Seventh Day Adventist<br>Protestant<br>Zionist<br>Ministries<br>Other) ___________(specify) Don't know<br>No response
105	How many biological children do you have?	|__] children<br>Don't know<br>No response
106	Do you want/ plan to have any more children?	Yes<br>No<br>Undecided<br>No response
107	Age (in completed years)	

406.	{Relevant if q402=2} ***RING USERS:*** Was a ring inserted during your visit today?	No<br>Yes, inserted by provider<br>Yes, inserted myself<br>No response

Sample week one check-in questions:

Number	Question	Response options
101.	**{relevant if (q006=2} PrEP RING USERS WHOSE RING WAS NOT INSERTED AT CLINIC:** Have you inserted the ring yet?	Yes<br>No<br>No response
102.	Why haven't you inserted the PrEP ring yet?<br><br>***Probe:*** Any other reasons?	***Risk***<br>No longer need PrEP (a)<br>***Personal***<br>Menses (c)<br>Medical issue (e.g. STI) (d)<br>Worried the ring will be uncomfortable (e)<br>Couldn't get it in right (f)<br>It fell out (g)<br>***Approval***<br>Have not discussed with partner (f)<br>Partner did not approve (g)<br>Family did not approve (h)<br>Judged by someone (i)<br>Afraid of being judged (j)<br>***Value***<br>Afraid of side effects (k)<br>Afraid not effective (l)<br>Afraid impact on fertility/pregnancy (m)<br>***Other***<br>Want to know more (t)<br>Other (specify: _______)(v)<br>No response

Sample follow-up questions:

Number	Question	Response options
501.	How easy or difficult is it to use PrEP ring?<br><br>***Read responses***	Very difficult<br>Somewhat difficult<br>Somewhat easy<br>Very easy
